# Phylogenetic analysis of torque teno virus genome from Pakistani isolate and incidence of co-infection among HBV/HCV infected patients

**DOI:** 10.1186/1743-422X-9-320

**Published:** 2012-12-28

**Authors:** Tabinda Hussain, Sobia Manzoor, Yasir Waheed, Huma Tariq, Khushbakht Hanif

**Affiliations:** 1 Atta-ur-Rehman School of Applied Biosciences, National University of Sciences and Technology, Islamabad, 44000, Pakistan

**Keywords:** Torque teno virus, TTV prevalence, Co- infection in hepatitis, Pakistani population, TTV genome

## Abstract

**Background:**

Torque Teno Virus (TTV) was the first single stranded circular DNA virus to be discovered that infects humans. Although there have been numerous reports regarding the prevalence of TTV from other countries of South Asia, there is severe lack of information regarding its prevalence in Pakistan. Thus the present study compiles the first indigenous report to comprehensively illustrate the incidence of the virus in uninfected and hepatitis infected population from Pakistan. Another aim of the study was to present the sequence of full length TTV genome from a local isolate and compare it with the already reported genome sequences from other parts of the world.

**Methods:**

TTV DNA was screened in the serum of 116, 100 and 40 HBV infected, HCV infected and uninfected individuals respectively. Nearly full length genome of TTV was cloned from a HBV patient. The genome sequence was subjected to in-silico analysis using CLC Workbench, ClustalW, ClustalX and TreeView. Statistical analysis was carried out in SPSS v17.0.

**Results:**

Our results report that 89.7%, 90.0% and 92.5% of HBV, HCV patients and healthy control population were positive for TTV infection. TTV genome of 3603 bp was also cloned from a local isolate and given the identity of TPK01. The TTV genome sequence mentioned in this paper is submitted in the GenBank/EMBL/DDBJ under the accession number JN980171. Phylogenetic analysis of TPK01 revealed that the Pakistani isolate has sequence similarities with genotype 23 and 22 (Genogroup 2).

**Conclusion:**

The results of the current study indicate that the high frequency of TTV viremia in Pakistan conforms to the reports from other areas of the world, wherever screening of TTV DNA was performed against 5^′^-UTR of the genome. The high sequence diversity among TTV genome sequences and the high frequency of prevalence makes it harder to study this virus in cellular systems.

## Background

Torque Teno Virus (TTV) is a circular ssDNA virus [1]. It has been classified in a newly characterized genus called Anelloviridae. The genome size of TTV varies from 3.6-3.8 kb [2].

TTV is unique in many ways, it is the first single stranded circular DNA virus to be discovered that infects humans [3]. Another distinguishing property of TTV is its small genome size for a virus that infects humans. Also the nucleotide sequence variation between genotypes of TTV is greater than 50% which explains its distribution to at least 39 genotypes [4]. Before the discovery of TTV, this high rate of mutation was never observed in a human virus with such a small genome size. Moreover TTV genome shows greater variation at ORF level than in the UTRs, which is again a distinctive trait limited to TTV and TTV-like viruses [5].

Reports regarding prevalence of TTV in blood show immense disparity, from a viremia rate of 2-23% [6] to its frequency in blood rising as high as 90% [7,8]. This variation in the rate of positive detection depends on the choice of target site of amplification in PCR, as the UTRs of the genome are more conserved as compared to the ORFs therefore primers from UTR will cross-match a large number of genotypes [9] and hence will give a higher rate of detection.

The conservation of sequence in the UTRs and divergence in ORFs translates to greater disparity at amino acid level than at the nucleotide level. For instance, the two TTV isolates SANBAN and TA278 share only 32% sequence identity at amino acid level while the nucleotide sequence of UTRs is 78% identical [10]. Protein expression of TTV indicates that three mRNAs are encoded from the three overlapping Open Reading Frames (ORFs) [11]. The exact function and location of these possible protein products is still under study [12].

As TTV was discovered in a non-A-G hepatitis patient, the prevalence of TTV has been repeatedly examined in subjects with hepatocellular carcinoma, Hepatitis B and C and at various stages of liver disease. Their results indicate a high rate (>90%) of prevalence both in healthy subjects and in patients with some form of liver disorder [13-16]. It is this omnipresence of TTV and the relative difficulty in propagating it in cell cultures, that makes the study of the TTV life cycle challenging.

In relation to hepatitis, some noteworthy observations have surfaced. In one study the histological grade score was higher in case of HCV-TTV co-infection as compared to infection with HCV only [17]. There have been claims that TTV viral load is a significant factor for hepatocellular carcinoma (HCC) in hepatitis C patients, irrespective of known risk factors [18]. In another study the examination of 25 pediatric cases revealed that children with co-infection of HBV and TTV showed higher histological activity index, periportal necrosis and portal inflammation scores [19].

More than 14 years have passed since the discovery of Torque Teno virus but the molecular pathway it follows in the host and its pathogenic role, if any, has yet to be uncovered. The only report regarding prevalence of the virus in Pakistan was made by Prescott *et al*., in 1999 [20]. Thus the present study compiles the first indigenous report to comprehensively illustrate the incidence of the virus in uninfected and hepatitis infected population from Pakistan. The genome of TTV was also cloned and sequenced. The sequence was analyzed and annotated through in-silico analysis and the phylogenetic relationship with other genotypes was determined.

## Results

### Prevalence of TTV detection by PCR

In this study 116, 100 and 40 samples respectively, of HBV, HCV infected and healthy individuals were screened with primers from 5’ UTR. The results are represented in Table 1. Average value of age and ALT levels along with the standard deviations and the male to female ratio of the study group are summarized in Table 2.

**Table 1:** Summary of TTV prevalence in different study groups

**Study group**	**No. of samples (N)**	**TTV positive(%age)**	**TTV negative (%age)**
HBV Patients	116	104(89.7%)	12(10.3%)
HCV Patients	100	90(90.0%)	10(10.0%)
Control Population	40	37(92.5%)	3(7.5%)

**Table 2:** Clinical features of the TTV positive and TTV negative cases

**Sample group**	**Mean age ± SD**	**Gender M:F ratio**	**Mean ALT Level ± SD**
Hepatitis B			
(N = 116)	34.1 ± 13.3	76:40	90.84 ± 47.7
TTV+	34.1 ± 12.56	69:35	93.35 ± 48.7
TTV-	34 ± 19	7:5	61.3 ± 13.56
Hepatitis C			
(N = 100)	38.72 ± 12.2	51:49	90.83 ± 40.6
TTV +	38.75 ± 11.4	42:48	90.86 ± 44.2
TTV-	38.4 ± 18.5	7:3	91.7 ± 39.2

Statistical Analysis proved that there was no significant relationship between factors such as age, gender and ALT levels of hepatitis patients and the respective prevalence of TTV in HBV/HCV infected and healthy populations. The p-values for each test were above 0.05 except in case of Fisher’s exact test for relationship between Age and TTV incidence where p-value was 0.048. Since the other two tests (t-test and Pearson’s chi-square) gave a value >0.05 therefore the result of Fisher’s exact was not sufficient to prove the hypothesis.

### Analysis of cloned TTV genome

The nearly full length TTV genome sequenced from Pakistan was named as TPK01 (GenBank accession number, JN980171). TPK01 isolate was 3,603 bp in length and had approximately 44 bp missing from the 5’ UTR and 126 bp missing from the 3’ UTR, comprising of the GC rich region, and due to primer constraints.

TPK01 was compared against the nucleotide repository of NCBI using Basic Local Alignment Search Tool (BLAST) [21] which returned the highest local alignment with isolate Kt-08f, genotype 22 (A/C No: AB054647), 90% query sequence matched with the subject with a maximum nucleotide identity of 77% and E-value of 0.0.

**Figure 1:**
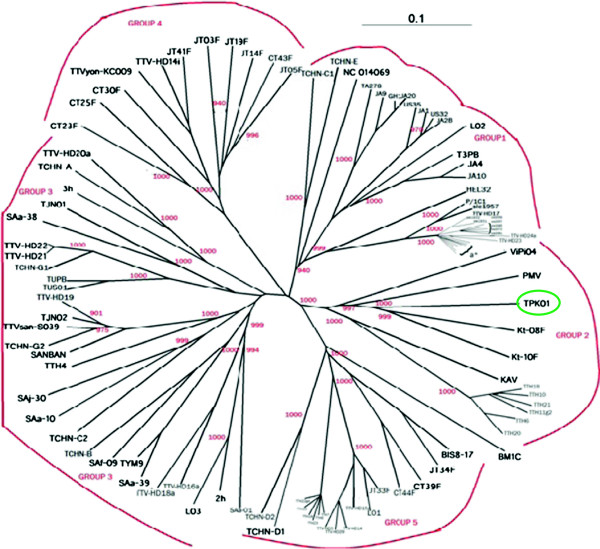
**Phylogenetic analysis of TTV TPK01 based on full-length genome and/or full length cds.** The tree was constructed using the NJ Algorithm included in the ClustalX (version 2.0.11). Bootstrap values of >900 out of 1,000 replicates are shown at the branch points. The tree was drawn using Treeview (version 1.6.6). TPK01 (highlighted in yellow) belongs to genogroup 2. The scale bar represents a 10% genetic difference.

TPK01 was compared with 106 other full-length genomes or full-length cds of TTV in order to obtain alignment for construction of the phylogenetic tree (Figure 1).

Phylogenetic analysis was able to conclude that our isolate matched most closely to the Genogroup 2 of TTV genotypes [22]. Other members of this group include, Kt-08f and Kt-10f from Indonesia [23], ViPi04 from Italy [24], PMV isolate from United Kingdom [25], KAV, TTH18, TTH10, TTH21, TTH11g2, TTH6, TTH20 isolates from Germany [26,27]. The NCBI Accession numbers of all sequences used in the phylogenetic analysis are indicated in Table 3. Due to the high sequence diversity between TTV genome sequences reported from around the world, the recommended method of classification is to assign them in broad genogroups rather than separate genotypes [28,29].

**Table 3:** Primers used for screening and cloning of TTV DNA

**S.No**	**Primer name**	**Purpose**	**Sequence**	**Position**	**Expected product size**
1.	T1F	Screening	5^′^--GTA AGT GCA CTT CCG AAT GGC TGA G-3^′^	91-115	133 bp
	(NG133)				
2.	T1R	Screening	5^′^--AGC CCG AAT TGC CCC TTG AC-3^′^	204-223	
	(NG132)				
3.	TTF2	Cloning	5^′^- TAT GTC GTC CAC TTC CTG GG-3^′^	50-69	3652 bp
4.	TTR2	Cloning	5^′^- GAG GAA GGA AGT CGG CCA TTT TG-3^′^	3684-3707	

The sequenced genome was annotated corresponding to the highest scoring hit in BLAST results i.e. Genotype 22 isolate Kt-08f (GenBank Accession No: AB054647) [23]. The ORF numbers were assigned according to Genotype 6 isolate HEL32 (GenBank Accession No: AY666122) [11].

**Figure 2:**
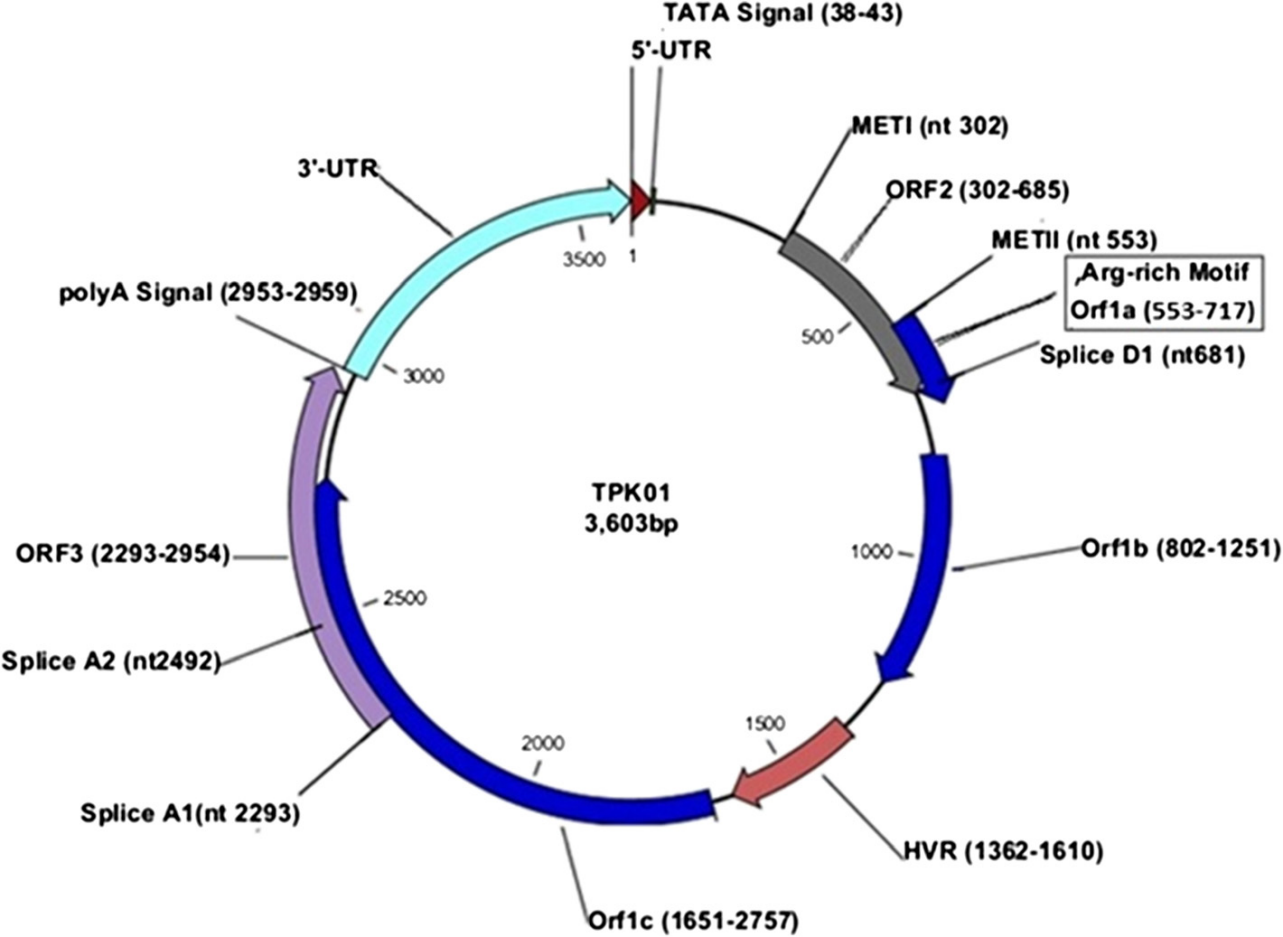
**Predicted annotation of TPK01.** Splice D, A1, A2, are the splice acceptor and donor sites. The ORF3 will be alternatively spliced to code for the putative proteins ORF2/3, ORF2/2, ORF1/2 and ORF1/1. HVR is the Hypervariable region found in TPK01. The sequence is shown as circular for convenience, although the GC-rich tract is missing from the 3’-UTR.

The demarcation of ORFs is similar to the Kt-08f isolate except for ORF1, which in this case is interrupted by premature stop codons at two positions. This could split ORF1 into three shorter reading frames namely; ORF1a, ORF1b, ORF1c. The pictorial view of TPK01 is given in Figure 2.

## Discussion

Torque Teno Virus (TTV) was first discovered in the blood of a post-transfusion non A-G hepatitis patient which led to TTV being repeatedly linked as the causal factor of cryptogenic hepatitis. There have been numerous reports about the TTV viremia in patients of Hepatitis B and C from all over the world but there is severe lack of data on epidemiology of TTV and its sequence analysis from Pakistan. Therefore the present study was designed to clarify the rate of prevalence of TTV in hepatitis infected population vis-à-vis healthy subjects, outline a relationship of TTV prevalence with the incidence of hepatitis and to finally analyze the full length genome sequence of TTV from Pakistani isolate.

The findings of this study report a TTV incidence rate of 89.7%, 90.0%, 92.5% in the Hepatitis B group, Hepatitis C group and control population respectively. Our statistical analysis refutes the hypothesis that the frequency of TTV incidence is influenced by the factors such as age, gender and ALT level in hepatitis patients.

The high prevalence of TTV alludes to the likelihood of a latent, possibly lifelong infection. Some of the patients in our study were less than 8 years of age. Our findings are complemented by other reports of TTV infection in infants and children [28,30-32] which provide evidence that TTV is a mother-to-child transmitted virus, as suggested by Yokozaki *et al*. (1999) rather than a blood-borne virus.

In any epidemiological study of TTV prevalence with a PCR based method, the design and target position of primers is crucial in determining the frequency of positive detection of virus. Due to the high sequence diversity among TTV genotypes it is improbable that a single set of primers will detect all types of TTV. For this reason the primers used in this study were chosen on the basis of extensive review of data pertaining to surveys of prevalence of TTV. It has been emphasized by the findings of multiple reports that primers from UTRs will have greater potential to detect multiple genotypes of TTV rather than those designed against the ORFs [33,34]. The reason being that there is higher sequence conservation in the non-coding regions than in the coding regions, where in some stretches of the UTRs more than 90% sequence identity has been observed [5,35].

Based on the above mentioned facts, for the present study primer pair NG133/NG132 [33] was selected. A thorough analysis of TTV detection strategies by Hu et al. suggests that a group of four primer sets have the ability to screen all genotypes of TTV and TTV-like viruses except genotype 21. Of this recommended array of primers, NG133/NG132 is a part [36].

The results of the present study are in parallel with data published from other parts of the world. For example, in the study mentioned above, the positive rate of TTV detection was found to be as high as 93% with 5’-UTR primers but only 10% with primers designed from ORF region in the healthy population of Japan. Another study which surveyed the TTV infection across different geographic locations (Japan, USA, Mayanmar, Korea, Nepal, Egypt, Bolivia, Ghana) revealed the frequency of positive result between 70-98% from UTR primers, both in healthy and disease infected population [16]. The only previous report of TTV infection rate from Pakistan was made in 1999 in blood donors from Aga Khan University, Karachi. The reported prevalence was only 16% with primers from the N22 region which belongs to the ORF1 of TTV [20]. In separate studies from Russia, Iran, and Hong Kong, infection rate in healthy population was 94%, 80% and 90% respectively [7,37,38]. In contrast to the above reports of high prevalence, TTV was observed in only 2.9% of healthy Iranian population emphasizing the fact that ORF primers have less conserved sequence which makes them incapable of matching multiple genotypes [6].

The only studies where the rate of infection in healthy population is relatively lower than our findings, when similar primers have been used, are from India and UAE [39,40]. In these studies nested PCR approach was followed where NG133/NG147 were used in the first round while NG134/NG132 amplified in the second round. Out of 50 healthy volunteers, only 26 tested positive which is a 52% infection rate. The same study shows that 80.6% of patients with fulminant hepatic failure and 76% of patients with Acute Viral Hepatitis tested positive for TTV infection. A different research that tested the prevalence in patients on hemodialysis and healthy subjects; the positive infection rate was 83% and 43% respectively [41]. The study from UAE [40] reported that 97.9, 95.7% of patients with HBV and HCV tested positive for TTV infection which aligns closely with our results of 89.7% and 90.0% of HBV and HCV patients testing positive for TTV infection. In contrast a mere 34.9% of healthy population was found to be carrier of TTV DNA which differs from our finding of 92.5% of control population that was observed as TTV positive.

The possible reasons for this marked variation in the frequency of infection in control population in our study and the above cited reviews could be the difference in the number of sample population, the fact that the prevalent genogroup in these areas is different from Pakistan or simply that TTV is more ubiquitous in Pakistan than in other countries.

The TTV isolate cloned in our study named TPK01 has sequence similarity spanning over most of the genome with the previously reported genotype 22 and 23 isolates Kt-08f and Kt-10f from Indonesia [23]. It is worth mentioning here that the previous isolates from Pakistan which were short clones of N22 region (200 bp approx.) belonged to genotype 1a in majority [20]. TPK01 also shows significant similarity to the PMV isolate from United Kingdom of genotype 17 [25]. Stretches of 5’-UTR of the PMV sequence are up to 92% identical to TPK01. Other sequences that show identity with TPK01 over some stretches of genome are ViPi04, isolate from Italy [24], tth6 and tth20 isolates of a Hodgkin’s disease patient from Germany [27]. The entries of genome that related most closely to TPK01 were all isolates belonging to genogroup 2.

The arrangement of ORFs, promoter and poly-A sites is similar to genotype 22 and other members of group 2. In TPK01 nt1362-1610 show no similarity with any of the TTV genomes assembled in NCBI Nucleotide database, this region corresponds to the HVR of ORF1 and has therefore hypermutated. In a study where TTV isolates were cloned from Hodgkin’s patients, the HVR extended over nt1425-1693 and showed 80 to 99% variation among the isolates that were cloned, whereas the overall variation in the sequence of the cloned TTV genomes was only 2% [27]. This high rate of variation that translates to divergence at the amino acid level could suggest immune-evasion by hypermutation [27,42]. Premature stop codons have also been observed in ORF1 of our isolate which splits the coding sequence into three shorter translation products. The occurrence of interrupted ORF1 has been observed in other TTV isolates as well. ORF1 splits in two potential reading frames in isolates tth7, tt32b8, tth8 while the isolate tth22g4 is interrupted twice which translates to three putative proteins [27]. TCHN-a, TCHN-c1, TCHN-d2, US32 also have premature stop codons which can give rise to two translation products instead of one [43,44].

In one study, subviral molecules could be isolated from a Hodgkin’s lymphoma cell line when it was transfected with full-length TTV genomes. It was found that additional splicing events and rearrangements within the genome raised ORFs that could not be explained by the parent genomic sequence. These subviral particles varied in sequence length from 172 bp to full length genome. Similar kinds of molecules were also isolated from sera of pregnant mothers [45]. These findings present the question that whether the multiple genotypes and types of TTV (Torque Teno Mini Virus, Torque Teno Midi Virus) are due to wide intra-genomic rearrangements or are actually separately evolved genotypes.

## Conclusion

The results of this study conform to the previous findings which have established the high rate of sequence variation in TTV genotypes. These variations in sequence are less pronounced in the UTR, but are seen extensively in the ORFs. This high level of sequence divergence and lack of apparent association with any disease poses serious questions about the role of this ever-present replicating and mutating form of virus. The hardest of all questions is the mode of translation and function of TTV proteins. Therefore it is inferred that TTV presents itself ubiquitously in human population irrespective of any disease association so far. The sequence variation in the genome of TTV can suggest rearrangements within the genome giving rise to new forms of TTV.

## Methods

### Sample collection

The study was reviewed and approved by the Ethics Committee of Atta-ur-Rahman School of Applied Biosciences, National University of Sciences and Technology, Sector H-12 (NUST), Islamabad, Pakistan. Informed consent from all patients and guardians of subjects below the age of 18 was obtained before proceeding with the study.

Blood samples were collected from subjects who came to Diagnostic Laboratory of Atta-ur-Rahman School of Applied Biosciences, NUST, Sector H-12, Islamabad, Pakistan for routine testing.

### Confirmation of HBV and HCV infection

The confirmation for Hepatitis B and C infection was done first with serological testing and then the positive results were further tested for the presence of HBV DNA and HCV RNA. The initial confirmation of infection with HBV was carried out with KHB EIA kits for anti-HB core, HBe/anti-HBe and HBsAg/anti-HBs whereas HCV infection was confirmed with KHB EIA kit for the detection of anti-HCV antibodies.

Further confirmation of HBV and HCV infection was carried out with RoboGene HBV DNA Quantification Kit which amplified a region of HBV surface gene while HCV RNA Quantification Kit was used to test against 5’-UTR of HCV genome. Standard manufacturer’s protocol was followed in all instances.

### Extraction of viral DNA

DNA was extracted from both HBV/HCV infected and uninfected control samples using NucleoSpin Blood MACHEREY-NAGEL DNA extraction kit. The DNA was eluted in 50 μl of Nuclease-free water.

### Primer designing

#### Screening primers

The primers for screening of TTV DNA (NG133 and NG132 as given in Okamoto *et al*., 1999b, [33]) were chosen on basis of their specificity and sensitivity of detection after extensive review of literature. The primers used in the study are listed in Table 4.

**Table 4:** Accession numbers and reference of 106 sequences used in the phylogenetic tree

**S No.**	**NCBI accession No.**	**Isolate name**	**Reference**
1.	AB017610	TA278	**Okamoto *et al*., 1998b**[8]
2.	AY823988	2 h	**Niel *et al*., 2005**[22]
3.	AY823989	3 h	**Niel *et al*., 2005**[22]
4.	AY666122	HEL32	**Qiu *et al*., 2005**[11]
5.	AB017613	TUS01	**Okamoto *et al*., 1999c **[33]
6.	AF261761	PMV	**Hallet *et al*., 2000**[25]
7.	AB054647	Kt-08f	**Muljono *et al*., 2001**[23]
8.	AB054648	Kt-10f	**Muljono *et al*., 2001**[23]
9.	AF435014	KAV	**Heller *et al*., 2001**[26]
10.	GU797360	BIS8-17	**Unpublished**
11.	DQ187006	BM1C-18	**Unpublished**
12.	AB064595	CT23F	**Peng *et al*., 2002**[28]
13.	AB064596	CT25F	**Peng *et al*., 2002**[28]
14.	AB064597	CT30F	**Peng *et al*., 2002**[28]
15.	AB064604	CT39F	**Peng *et al*., 2002**[28]
16.	AB064598	CT43F	**Peng *et al*., 2002**[28]
17.	NC014075	CT44F	**Peng *et al*., 2002**[28]
18.	AF122913	GH1	**Mushahwar *et al*., 1999**[3]
19.	AF122916	JA1	**Erker *et al*., 1999**[5]
20.	AF122918	JA2b	**Erker *et al*., 1999**[5]
21.	AF122917	JA4	**Erker *et al*., 1999**[5]
22.	AF122915	JA9	**Erker *et al*., 1999**[5]
23.	AF122919	JA10	**Erker *et al*., 1999**[5]
24.	AF122914.3	JA20	**Erker *et al*., 1999**[5]
25.	AB064599	JT03F	**Peng *et al*., 2002**[28]
26.	AB064600	JT05F	**Peng *et al*., 2002**[28]
27.	AB064601	JT14F	**Peng *et al*., 2002**[28]
28.	AB064602	JT19F	**Peng *et al*., 2002**[28]
29.	AB064606	JT33F	**Peng *et al*., 2002**[28]
30.	NC_014076	JT34F	**Peng *et al*., 2002**[28]
31.	AB064603	JT41F	**Peng *et al*., 2002**[28]
32.	AY026465	L01	**Liu *et al*., 2002**[43]
33.	AY026466	L02	**Liu *et al*., 2002**[43]
34.	AF371370	L03	**Liu *et al*., 2002**[43]
35.	NC014069	NC014069	**Unpublished**
36.	AF298585	P1C1	**Unpublished**
37.	AB060597	SAa-01	**Okamoto *et al*., 2001**[23]
38.	AB060594	SAa-10	**Okamoto *et al*., 2001**[23]
39.	AB060593	SAa-38	**Okamoto *et al*., 2001**[23]
40.	AB060592	SAa-39	**Okamoto *et al*., 2001**[23]
41.	AB060596	SAf-09	**Okamoto *et al*., 2001**[23]
42.	AB060595	SAj-30	**Okamoto *et al*., 2001**[23]
43.	AB025946.2	SANBAN	**Hijikata *et al*., 1999**[40]
44.	AM712003	Sle1931	**Leppik *et al*., 2007**[45]
45.	AM712004	Sle1932	**Leppik *et al*., 2007**[45]
46.	AM711976|	Sle1957	**Leppik *et al*., 2007**[45]
47.	AM712030	Sle2057	**Leppik *et al*., 2007**[45]
48.	AM712031	Sle2058	**Leppik *et al*., 2007**[45]
49.	AM712033	Sle2061	**Leppik *et al*., 2007**[45]
50.	AM712034	Sle2065	**Leppik *et al*., 2007**[45]
51.	AM712032	Sle2072	**Leppik *et al*., 2007**[45]
52.	AF247138	T3PB	**Biagini *et al*., 2000**
53.	AF345526	TCHN-A	**Luo *et al*., 2002**[44]
54.	AF348409	TCHN-B	**Luo *et al*., 2002**[44]
55.	AF345523	TCHN-C1	**Luo *et al*., 2002**[44]
56.	AF345527	TCHN-C2	**Luo *et al*., 2002**[44]
57.	AF345524	TCHN-D1	**Luo *et al*., 2002**[44]
58.	AF345525	TCHN-D2	**Luo *et al*., 2002**[44]
59.	AF345522	TCHN-E	**Luo *et al*., 2002**[44]
60.	AF345528	TCHN-F	**Luo *et al*., 2002**[44]
61.	AF345521	TCHN-G1	**Luo *et al*., 2002**[44]
62.	AF345529	TCHN-G2	**Luo *et al*., 2002**[44]
63.	AB028668	TJN01	**Ukita *et al*., 2000**[46]
64.	AB028669	TJN02	**Ukita *et al*., 2000**[46]
65.	AJ620218	TTH3	**Jelcic *et al*., 2004**[27]
66.	AJ620226	TTH4	**Jelcic *et al*., 2004**[27]
67.	AJ620227	TTH5	**Jelcic *et al*., 2004**[27]
68.	AJ620212	TTH6	**Jelcic *et al*., 2004**[27]
69.	AJ620230	TTH7	**Jelcic *et al*., 2004**[27]
70.	AJ620231	TTH8	**Jelcic *et al*., 2004**[27]
71.	AJ620219	TTH9	**Jelcic *et al*., 2004**[27]
72.	AJ620213	TTH10	**Jelcic *et al*., 2004**[27]
73.	AJ620214	TTH11	**Jelcic *et al*., 2004**[27]
74.	AJ620232	TTH13	**Jelcic *et al*., 2004**[27]
75.	AJ620228	TTH14	**Jelcic *et al*., 2004**[27]
76.	AJ620220	TTH16	**Jelcic *et al*., 2004**[27]
77.	AJ620221	TTH17	**Jelcic *et al*., 2004**[27]
78.	AJ620215	TTH18	**Jelcic *et al*., 2004**[27]
79.	AJ620233	TTH19	**Jelcic *et al*., 2004**[27]
80.	AJ620216	TTH20	**Jelcic *et al*., 2004**[27]
81.	AJ620217	TTH21	**Jelcic *et al*., 2004**[27]
82.	AJ620234	TTH22g4	**Jelcic *et al*., 2004**[27]
83.	AJ620235	TTH23	**Jelcic *et al*., 2004**[27]
84.	AJ620222	TTH25	**Jelcic *et al*., 2004**[27]
85.	AJ620223	TTH26	**Jelcic *et al*., 2004**[27]
86.	AJ620224	TTH27	**Jelcic *et al*., 2004**[27]
87.	AJ620229	TTH29	**Jelcic *et al*., 2004**[27]
88.	AJ620225	TTH31	**Jelcic *et al*., 2004**[27]
89.	FR751471	TTV-HD14i	**de Villiers *et al*., 2011 **[47]
90.	FR751472	TTV-HD15a	**de Villiers *et al*., 2011 **[47]
91.	FR751476	TTV-HD16a	**de Villiers *et al*., 2011 **[47]
92.	FR751488	TTV-HD17	**de Villiers *et al*., 2011 **[47]
93.	FR751489	TTV-HD18a	**de Villiers *et al*., 2011 **[47]
94.	FR751491	TTV-HD19	**de Villiers *et al*., 2011 **[47]
95.	FR751492	TTV-HD20a	**de Villiers *et al*., 2011 **[47]
96.	FR751498	TTV-HD21	**de Villiers *et al*., 2011 **[47]
97.	FR751499	TTV-HD22	**de Villiers *et al*., 2011 **[47]
98.	FR751500	TTV-HD23a	**de Villiers *et al*., 2011 **[47]
99.	FR751506	TTV-HD24a	**de Villiers *et al*., 2011 **[47]
100.	AB038620	TTVsan-s039	**Takahashi *et al*., 2000 **[48]
101.	AB038621	TTVyon-Kc009	**Takahashi *et al*., 2000 **[48]
102.	AF247137	TUPB	**Biagini *et al*., 2000 **[49]
103.	AB050448	TYM9	**Okamoto *et al*., 2000 **[50]
104.	AF122921	US32	**Erker *et al*., 1999**[5]
105.	AF122920	US35	**Erker *et al*., 1999**[5]
106.	DQ361268	ViPi04	**Maggi *et al*., 2006**[24]

#### Cloning primers

Primers were designed manually for cloning of Torque Teno virus genome. A total of 23 sequences of full-length or nearly full-length TTV genomes retrieved from NCBI nucleotide database were aligned in CLC Workbench 6.1.1 [51], the primers were designed from relatively conserved portions.

#### Amplification of TTV DNA

In order to clone the TTV genome, the reaction mixture constituted of 5 μl of extracted DNA, 2.5 U each of DreamTaq Polymerase (Fermentas EP0702) and Long PCR Enzyme Mix (Fermentas K0182), 10pmol each of forward and reverse primer, 2 m*M of* dNTPs. The PCR mixture was preheated at 95°C for 5 minutes, then run 35 cycles with these parameters: 95°C for 40 seconds, 60°C for 40 seconds, 68°C for 4 minutes, and the final extension was at 68°C for 10 minutes in an AB-Veriti 96-well Thermocycler.

For the detection of TTV DNA in HBV/HCV infected population and healthy controls the reaction mixture constituted of 8 μl of extracted DNA, 1.5 U of Taq Polymerase, 10 pmol of forward and reverse primer and 2 mM dNTPs. The PCR mixture was preheated at 95°C for 3 minutes, then run 35 cycles with these parameters: 95°C for 20 seconds, 60°C for 40 seconds, 72°C for 30 seconds, and the final extension was at 72°C for 7 minutes in a AB-Veriti 96-well Thermocycler. With every batch of screening reactions, a positive and negative control was used to eliminate the chances of any false negative results. A positive control was the serum sample which was confirmed for TTV infection after sequencing while the negative control is a serum sample repeatedly tested with multiple sets of primers from various parts of the TTV genome but returned negative result in all cases (Detail of primers is provided in Additional file 1).

A positive result meant the amplification of a 133 bp fragment from T1F/TIR primers. The positive and negative control were also loaded on gel along with the gene ruler.

#### Cloning of TTV genome

Due to high sequence diversity within the TTV genome, multiple sets of primers were used to amplify the full length genome. The TTV genome was successfully amplified from a HBV positive subject.

The gel eluted 3.6 kb amplified TTV product was cloned in a pCR 2.1 vector using TOPO TA Cloning Kit (Catalog no: K4500-01, Invitrogen, USA). The ligated vector was then transformed in Top10 strain of *Escherichia coli*. Recombinant plasmids were then prepared from cell cultures. Initially, the plasmid was confirmed for the presence of TTV genome insert with restriction digestion.

#### Sequencing

The cloned TTV genome was sequenced with M13 universal primers as well as two sets of internal primers (Details provided in Additional file 1) using an ABI 3730×l DNA Sequencer (Biobasic Inc Canada).

#### In-silico analysis

Multiple genome sequences available in NCBI database were retrieved and aligned with the local Pakistani isolate in CLC Workbench 6.1.1 and ClustalX, version 2.0.11 [52]. Phylogenetic tree was then constructed using Neighbour-Joining Algorithm included in ClustalX. The robustness of the tree was tested with bootstrapping technique where the tree was replicated 1000 times with a seed value of 111. TreeView, version 1.6.6 was used to draw the final tree. The protein products from the ORFs were predicted in CLC Workbench 6.1.1. Alignment with pre-reported proteins was done in CLUSTALW and CLC Workbench. Scores for amino acid identity and similarity was retrieved from SIAS (Sequences Identities and Similarities) server [53].

#### Statistical analysis

The prevalence data generated with the screening of TTV DNA in HBV/HCV and healthy population was analyzed with statistical tests carried out in SPSS, version 17.0. The hypothesis to be tested was whether factors such as age, gender and ALT levels of hepatitis patients affect the rate of prevalence of TTV. Student’s t-test, Pearson’s correlation and chi-square/fisher’s exact test were applied wherever relevant. P value <0.05 was considered significant.

## Competing interests

The authors hereby declare that they have no conflicts of interest.

## Authors’ contributions

SM and TH conceived the study. TH performed all the experiments, designed the primers and did the analysis. TH searched the literature and drafted the manuscript. YW, KH, HT helped in sample collection and data analysis. SM critically reviewed the manuscript. SM supervised and designed the study, gave final approval and helped TH in performing the work. All authors have read and approved the final version of the manuscript.

## Supplementary Material

Additional file 1 Primers used for establishing negative control and sequencing. Click here for file
