# Supplementary material for: Phylogenetic analysis of torque teno virus genome from Pakistani isolate and incidence of co-infection among HBV/HCV infected patients
Source: Virol J. 2012 Dec 28;9:320. doi: 10.1186/1743-422X-9-320 (PMC3573928; doi:10.1186/1743-422X-9-320)
Supplement: Additional file 1 — Primers used for establishing negative control and sequencing. [file 1743-422X-9-320-S1.doc]

| **S.No** | **Primer Name** | **Purpose** | **Sequence** | **Position**  **(According to TA278)** | **Expected Product Size** |
| --- | --- | --- | --- | --- | --- |
| 1. | T2F | Screening | 5′- AGT TTT CCA CGC CCG TCC GCA GC -3′ | 114-136 | 120bp |
| 2. | T2R | Screening | 5′- GCC AGT CCC GAG CCC GAA TTG CC-3′ | 211-233 |
| 3. | NIF | Screening | 5`-GCT ACG TCA CTA ACC ACG TG-3` | 6-25 | 199bp |
| 4. | N1R | Screening | 5`-CTT CGG TGT GTA AAC TCA CC-3` | 204-185 |
| 5. | T3F | Screening | 5`-CGA AAG TGA GTG GGG CCA GAC TTC-3` | 3337-3360 | 403bp |
| 6. | T3Rb | Screening | 5`-AAA GAG GAA GGA AGT CAG CC-3` | 3690-3709 |
| 7. | C2F | Screening | 5`-ACC ACA CAA ACT ATA GCC CA-3` | 1495-1514 | 144bp |
| 8. | C2R | Screening | 5`-GGT GCC TGG ATA TGC ATA AG-3` | 1619-1638 |
| 9. | C3Fa | Screening | 5′- ACA GAC AGA GGA GAA GGC AAC ATG-3′ | 1900-1923 | 286bp |
| 10. | C3R | Screening | 5′-CTG GCA TTT TAC CAT TTC CAA AGT T-3′ | 2161-2185 |
| 5. | THS1F | Sequencing | 5′ -TGT GGA ACG GGC ACA GTC-3′ | - | - |
| 6. | THS1R | Sequencing | 5′-CTC GGA GTC CGT TTA ATA AAG C -3′ | - | - |
| 7. | TH2SF | Sequencing | 5′- GCT CAC CAC AAA CTG ACA AC-3′ | - | - |
| 8. | TH2SR | Sequencing | 5′-CGT AGT CTT CTC CTT GCA GC- 3′ | - | - |

Primers used for establishing negative control and sequencing
